# Regulation of Immunity via Multipotent Mesenchymal Stromal Cells

**Published:** 2012

**Authors:** Y. P. Rubtsov, Y. G. Suzdaltseva, K. V. Goryunov, N. I. Kalinina, V. Y. Sysoeva, V. A. Tkachuk

**Affiliations:** Faculty of Fundamental Medicine, Lomonosov Moscow State University; Institute of Experimental Cardiology

**Keywords:** immune system, multipotent mesenchymal stromal cells, inflammation, autoimmune disease, regeneration, immune suppression

## Abstract

Immune cells responsible for inflammation development are involved in tissue damage caused by wounding and various pathologies. Control of immune cell activation could be of significant benefit for regenerative medicine and the treatment of patients with autoimmune and degenerative diseases. It is a proven fact that MCSs (multipotent mesenchymal stromal cells) are capable of suppressing immune responses via the inhibition of dendritic cell maturation and via the restraining of the T, B, and NK cell function in the course of autoimmune diseases and various forms of inflammation. MSCs can be isolated easily from almost every type of tissue or organ and subsequently expanded*in vitro*. These cells are self-renewable and can be differentiated into various cell types of mesenchymal lineage. The current review contains a collection and critical analysis of data regarding the molecular mechanisms responsible for cross-talk between immune cells and MSCs. Some of these mechanisms can be used for the development of new practical approaches for the treatment of autoimmune diseases.

## Introduction: General characteristics of msc

Multipotent mesenchymal stromal cells (MSCs) were originally characterized in the pioneering study of Friedenstein *et al.* in 1971 [1]. It was shown in that study that a heterogeneous fraction of cells bearing morphological resemblance to fibroblasts can adherently grow in a culture, tolerate numerous passages, and be isolated from bone marrow cells. MSCs express a set of markers on their surface (suggesting their mesenchymal origin) and are capable of differentiating into adipose, bone, and cartilage cells [1] and, to a lesser extent, into other cell types. The set of markers characteristic of MSCs includes CD105, CD166, CD54, CD90, CD55, CD13, CD73, Stro-1, and CD44; meanwhile, the surface of an MSC does not contain the hematopoietic markers CD14, CD45, CD34 and СD133 [2]. It was subsequently ascertained that cells with similar properties can be isolated not only from the bone marrow, but also from other sources (in particular, from adipose tissue) [3].

A detailed study of the properties of MSCs has demonstrated that self-sustaining clones can be derived from a fraction of single cells [4]. MSC populations from different sources can be passaged, as opposed to terminally differentiated cells; culture heterogeneity is strongly passage-dependent [5]. The rates of growth and division of MSCs in a culture gradually decrease due to telomere shortening at chromosome ends [6, 7].

The absence of any “reliable” surface markers renders the *in vivo* identification and study of MSCs extremely difficult; therefore, we have yet to determine whether MSCs are an artifact of *in* *vitro* isolation and cultivation of a complex cell mixture, or whether indeed this population exists in the organism. Opinions concerning the nature of MSC differ considerably. It has been clearly demonstrated in a number of studies that MSCs resemble fibroblasts (another stromal cell type) in terms of many characteristics [8]. The authors of a number of studies compare MSCs with the population of pericytes; i.e. vascular endothelium-associated cells that carry a set of markers on their surface, differing from that in MSCs to only a small extent [9, 10]. Nevertheless, the interest of researchers and medical investigators in MSCs is primarily a result of the unique properties of MSCs, which make these cells a promising object for cell and gene therapy; issues of their origin and philogeny ultimately fade into insignificance.

## MSCs MIGRATE TO THE LESION LOCUS

When transplanted into animals with induced lesions or internal pathologies, MSCs are capable of migrating to the lesion site or to the inflammation focus. This discovery was confirmed by the results of experiments devoted to the systemic transplantation of variously labelled cells into recipients with the above-mentioned lesions (fluorescent protein-expressing cells were used, cells from male donors were transplanted into female recipients, human cells were used for heterologous transplantation into mice or rats) [11–15]. After a short period of time, the transplanted cells can be detected at the lesion site. MSC migration to the lesion (inflammation) site depends on chemokines, which is indirectly evidenced by the results of an analysis of chemokine receptor expression by MSCs. These cells express a wide range of chemokine receptors [16–18]. The contribution of most of them to the directed migration of MSCs has not yet been ascertained; however, it has been shown that SDF-1 and its receptor called C-X-C chemokine receptor type 4 (CXCR4) play the key role in this process. The CXCR4 level increases significantly in cells under stress conditions [16, 19, 20]. Disruption of signaling through this receptor using biochemical or genetic methods impairs MSC migration to the lesion/inflammation sites [19]. CXCR4 plays an essential role, since this receptor is also responsible for the retention of the hematopoietic stem cells in the bone marrow. Stem cells may leave the bone marrow as a result of systemic lesions due to the competition between MSCs and hematopoietic cells for the CXCR4 ligand – SDF-1 [21, 22]. For some time it was believed that MSC migration to the damaged tissue was indicative of active participation of these cells in tissue repair and regeneration. Additional studies of the behavior and migration of MSCs upon heterological transplantation clearly show that the proportion of MSCs that reach the lesion site post-transplant is very low. Moreover, the cells do not remain in the tissue and soon disappear. In this context, the initial assumption that the major role of MSCs was the direct replacement of the damaged-tissue cells through differentiation was dismissed[10]. Instead, the hypothesis that MSCs can facilitate the division and differentiation of stem and precursor cells, thus regulating their recruitment and survival upon stress conditions and injuries by secreting soluble factors, was proposed [23]. Therefore, it was suggested that MSCs serve as a mobile supplier of the factors necessary for tissue repair and regeneration.

## SECRETORY POTENTIAL OF MSCs AND REGENERATIVE PROCESSES

**Fig. 1:**
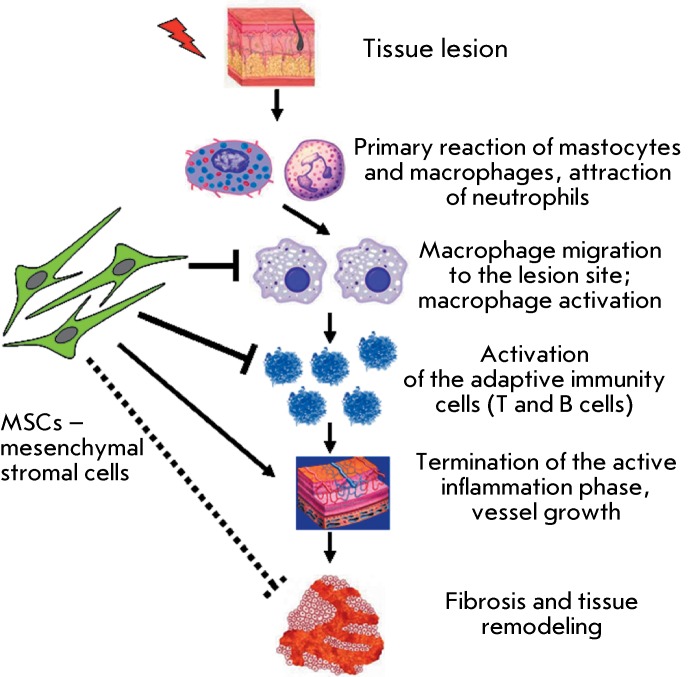
Main events following damage to inflammatory/wound tissue and the involvement of immune cells. The effect of MSCs on particular steps is shown with arrows in the case of positive influence; and with blunt-end arrows, in the case of negative (inhibitory) influence.

MSCs possess a unique property, which is secretion of a wide range of biologically active molecules, such as growth factors, cytokines, hormones, and low molecular weight mediators, which regulate the key physiological processes [23]. Factor production and the ability to produce/destroy the cell matrix underlie the physiological effect that MSCs have on the damaged tissue [24–26]. It has been demonstrated that the production of soluble factors by MSCs can support tissue cells, in addition to resident stem and precursor cells under inflammatory conditions and hypoxia, which inevitably accompany wound and pathological lesions [27–29]. It has been proven that the secretion of proangiogenic factors, such as VEGF, IGF-1, etc., by MCSs accelerates vascular growth and maturation at the lesion site [30–32]; the secretion of neurotrophic factors (in particular BDNF) facilitates the recovery of damaged neurons [33–35]; and the secretion of morphogenic proteins of the TGF-β family facilitates bone and cartilage tissue repair after a fracture [36–38] ( *Fig. 1* ). It is very likely that immediate contact with the surrounding cells and structures (microenvironment) also plays a significant role in the regenerative function of MSCs; however, few experimental studies exist to support this idea.

## Immune cells in tissue damagE and regeneration

When discussing the specific conditions accompanying tissue healing and repair processes, specific attention should be focused on the contribution of immune cells. It is a known fact that the immune system of mammals, including the human immune system, is a complex protective mechanism consisting of numerous types of cells that fight against infectious agents of different origin. The oldest immunity segment in terms of its evolution is represented by the cells responsible for recognizing foreign molecules and providing an immediate response to their presence [39]. These cells use molecular signalling to “pass the baton” to the adaptive immunity cells responsible for the development of the powerful immune response that is typically accompanied by the release of significant amounts of cytotoxic and proinflammatory molecules [40, 41]. Unfortunately, it is not easy to control this powerful and complex mechanism and accurately measure the adequate strength and direction of the attacks. The immune response is accompanied by acute or chronic damage to tissues and organs [39].

There exists a quite definite order of immune system reactions that accompany any damage to internal organs, injury, or infection. Tissue-resident mast cells, dendritic cells (DC), and macrophages act as damage sensors [38]. They initiate the cascade of immune reactions via the release of proinflammatory cytokines, chemokines, and factors that facilitate the migration and stimulation of other cell types. Cytokines and adhesion molecules, which ensure rapid neutrophil accumulation at the lesion site, play the key role in this process [39]. In turn, cytokine and chemokine production by neutrophils causes macrophage migration and the release of additional proinflammatory cytokines, such as IFN-γ and TNF-α [40]. The secretion of larger amounts of inflammatory cytokines recruits T and B cells by accelerating their activation and maturation. These cells accumulate at the lesion site, thus enhancing inflammation due to the production of new doses of cytokines and proinflammatory factors, often resulting in an undesired lesion and subsequent cell death in the surrounding tissue [41–44]. In turn, the inflammatory response initiates the molecular mechanisms that suppress activation and division of immune system cells. These mechanisms include an increase in sensitivity of the activated cells to apoptosis, an upregulation of the expression of anti-inflammatory cytokine (IL-10 and TGF-β) receptors on the surface of immune cells, production of these cytokines by activated cells, the elevated production of negative coactivator molecules, the activation of regulatory cells and an increase in their number [45–47]. All these events result in the completion of the acute phase of the immune response, the death of injured and activated cells, and the phagocytosis of dead cells and their fragments by professional phagocytes [48]. Meanwhile, the production of such factors as TGF-β causes fibrotic changes in the tissue structure and facilitates the replacement of the original tissue with fibrin and connective tissue [49, 50]. A significant role belongs both to the cells of the surrounding tissue and to vascular endothelial cells, which by secretion and release of factors from the extracellular matrix direct the migration of particular effector cells into the lesion site [51]. To summarize, it should be emphasized that cells of the immune system are involved into all phases of the regenerative processes in tissues ( *Fig. 1)* . The participation of these cells actually defines the timeline and efficiency of the healing. Furthermore, the level of tissue inflammation and lesion considerably depends on the interaction between tissue cells and cells of the immune system.

## MSC ANTIGEN PRESENTATION

Taking into account the secretory potential of MSCs and the effect on the microenvironment at the lesion site, the positive effect of MSCs in different models of tissue regeneration can (at least to some extent) be accounted for by their influence on cells of the immune system ( *Fig. 1* ). In this context, the immunological properties of MSCs have been studied rather thoroughly. Unfortunately, this does not apply to the molecular mechanisms being responsible for these properties. In immunological terms, MSCs strongly differ from body cells by their almost complete inability to be recognized by the immune system due to their phenotypic features [52, 53]. As a result of this property, MSCs are a promising object for application in transplantology, since it allows one to bypass the problem of immunological compatibility. In comparison with other cell lineages, MSCs express an extremely insignificant amount of MHC I and MHC II molecules and carry no costimulatory molecules CD40, CD80, or CD86, which are required for T cell activation [54]. Meanwhile, MHC expression recovers during the differentiation, resulting in the recognition and destruction of the MSC progeny by the recipient’s immune system cells [55]. MSCs do not cause allogeneic mixed lymphocyte reaction in completely heterologous cultures [54]. MSC-mediated expression of MHC may vary depending on culturing conditions. In particular, MSCs activate the expression of MHC genes in the presence of small IFN-γ concentrations, which results in their capability of antigen presentation ( *in vitro* ). High doses of IFN-γ do not have this effect [56].

**Fig. 2:**
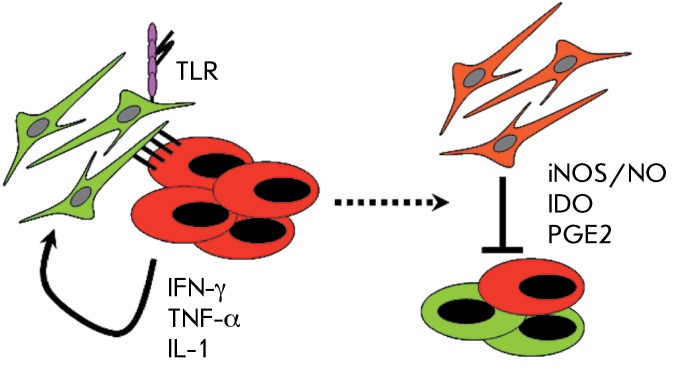
Schematic representation of the key factors involved in immunosuppression stimulation by MSC (on the left) and soluble effector molecules mediating the inhibitory effect of MCS on T cell function (on the right).

It has recently been demonstrated that MSCs can suppress the immune response by inhibiting NK maturation, suppressing the functions of T and B lymphocytes and natural killer (NK) cells [57–60].

## MSC IMMUNOREGULATION *IN VITRO*

**Fig. 3:**
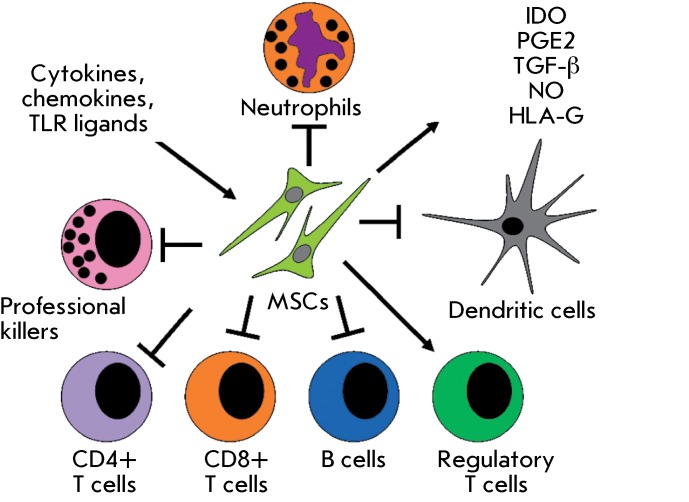
Spectrum of MSC-mediated immunosuppression cellular targets. MSC immunosuppression inducers are presented in the frame on the left-hand side, the main molecules – mediators of suppression – on the right-hand side. MSCs induce neutrophil apoptosis, inhibit dendritic cell maturation and secretion of proinflammatory cytokines (IFN-γ, IL-12, TNF-α), slow down proliferation and B-cell differentiation towards plasma cells, decrease immunoglobulin secretion, limit division of NK, CD4 and CD8 T cells, and limit the secretion of proinflammatory cytokines and the maturation of cytotoxic T cells from CD8 T cells. At the same time, MSCs stimulate IL-10 production by dendritic and regulatory T cells and boost expansion of regulatory T cells. The arrows indicate the positive effect of MSCs on cell function, whereas the blunt-end arrows indicate the negative effect of MSCs.

Most of the data on the immunological properties of MSCs has been obtained as a result of experiments on *in vitro* cocultivation or the joint incubation of MSCs and cells of the immune system. In these types of experiments, human blood leukocytes, or individual populations (e.g., T cells), were placed into the MSC-containing culture following activation. The effect of MSCs on the immune cells or, vice versa, the effect of immune cells on MSCs was then determined by measuring the cell division rate, the metabolic activity, the level of activation marker expression, the apoptosis level, and the secretion of cytokines and growth factors, etc. The following major regularities and mechanisms which have an impact on the results of the interaction between MSCs and cells of the immune system have been revealed [57–60] ( *Fig. 2, 3* ). It turns out that MSCs have different effects on different types of cells of the immune system. Naive (non-activated) T cells survive and divide in culture better in the presence of MSCs and MSC culture supernatants. Meanwhile, the activated T cells are susceptible to immunosuppression in the presence of MSCs. It has been ascertained that MSCs reduce the proliferative potential of T cells, the expression of activation markers and coactivatory molecules, and their ability to secrete proinflammatory cytokines, such as IFN-γ and TNF-α [58, 59, 61]. A similar effect was also observed for dendritic cells. After coculturing human or murine dendritic cells with MSCs, with DC maturation characterized by the expression of the molecules of the major histocompatibility complex on the cell surface, the capability of processing and representing protein antigen peptides to CD4 and CD8 T cells decreased in comparison to the control cocultures [60, 62, 63]. The effect also consisted in the reduction of the level of costimulatory molecules required for productive antigene presentation for T cells. Moreover, MSCs have a negative impact on the activation of immune cells of other types (in particular, NK [64, 65] and B cells [57, 66, 67]) in a culture. Inhibition of division and secretion of various immunoglobulins (IgA, IgM, IgG), as well as a decrease in chemokine receptor (CXCR4, CXCR5, CXCL12) expression manifesting itself in the suppression of cell chemotaxis, is observed for B cells [57, 64]. A set of factors secreted by MSCs have a negative impact on antigen production by plasma cells as a result of the activity of the CCL2 and CCL7 ligands that are formed as a result of the activity of matrix metalloproteinases being released from MSCs [65] ( *Fig. 3* ).

In early studies, the influence of MSCs on immune cells was determined in a blood mononuclear cell culture activated by preliminary incubation with antibodies against a T cell receptor or with nonspecific activators of the immune response (hemagglutinin, superantigens) [57–60]. For this assays T cells are the most convenient cell population, since it is the most abundant and the best characterized fraction of cells of the immune system. It is for this reason that the mechanism of the MSC effect on T cells has been studied appreciably well. It has been ascertained from the experiments on the MSC effect on activation and the effector function of T cells that only MSCs that were pre-incubated with activated T cells display immunosuppressive properties [68] ( *Fig. 2* ). Furthermore, incubation of MSCs with individual, purified proinflammatory cytokines (e.g., with IFN-γ) results in the emergence of these properties in MSCs (and MSC culture supernatants) [69–72]. This fact implies that cytokines stimulate MSCs, and this “activation” underlies the manifestation of immunosuppressive properties by MSCs ( *Fig. 2* ).

## ACtivation of immunosuppressIve properties of Msc requires preliminary stimulation of msc with proinflammatory cytokines

Which cytokines are critical for the manifestation of MSC’s immunosuppressive properties? The answer to this question has been obtained using blocking antibodies against various proinflammatory cytokines in cocultures of MSCs and activated T cells [69–72]. The use of this approach has demonstrated that the neutralization of IFN-γ, and decrease in the level of the IFN-γ receptor by the over-expression of microRNAs in MSCs, which interfere with the mRNA of one of its subunits, and the use of MSCs from IFN-γ receptor knockout mice result in a considerable reduction in the ability of these modified MSCs to suppress T-cell activation in a culture [69]. An alternative pathway for MSC activation by proinflammatory cytokines requires simultaneous participation of several proteins, in particular IFN-γ, TNF-α, and IL-1β. The requirement in these cytokines has been confirmed in *in vitro * experiments with blocking antibodies to the corresponding cytokines. It is worth noting that the blockage of any one or two different cytokines (pairwise) allowed a negligible restriction of the immunosuppressive properties of MSCs in the culture [69]. Only the simultaneous blocking of all three factors resulted in a pronounced physiological effect.

## MOLECULAR MECHANISMS OF MSC-MEDIATED IMMUNOREGULATION

It has been demonstrated, by a study of the molecular differences between “regular” and activated MSCs, that the expression of a number of genes, controlling suppression mechanisms, is triggered after treatment of MSCs by cytokines ( *Fig. 2* ). In particular, the level of indolamine-2,3-dioxygenase (IDO) in MSCs increases as a result of the action of proinflammatory cytokines [73]. It was revealed in earlier studies that IDO is a negative regulator of the T cell function. The secreted form of this enzyme is believed to diminish the level of free tryptophan (rapidly dividing activated T cells require large amounts of this amino acid) [74]. Moreover, tryptophan catabolite kynurenine, which is a product of IDO enzyme activity, also suppresses T-cell activation [74]. The experiments, in which a synthetic IDO inhibitor or MSCs from IDO-deficient mice was used, lend further credence to the significant role of this protein in MSC-mediated immunosuppression [69, 74, 75].

An alternative pathway of MSC activation based on simultaneous stimuli from IFN-γ, TNF-α, and IL-1β has also been ascertained at the molecular level and relies mainly on a considerable increase in the expression of the *iNOS * (inducible NO synthase) gene by MSCs. iNOS is an enzyme responsible for NO production by cells under stress conditions. The level of *iNOS* gene transcription under normal conditions is extremely low. The level of *iNOS * is known to significantly increase in many cells of the immune system under the action of cytokines and other stress factors [76]. An increase in the level of iNOS in MSC upon activation may attest to the fact that these cells enhance NO production. According to the existing data, the effect of NO on stimulated T cells consists in the suppression of cell division, cytokine secretion, and presumably, in an increase in the level of cell death. It has been shown by using inhibitors and iNOS-deficient MSCs that iNOS or NO activity is required for MSCs to be able to manifest their immunosuppressive properties [76].

It is interesting to note that recently obtained data appears to indicate that various immunosuppressive mechanisms may depend on the presence/absence of intercellular contacts. In the case of contact cocultivation of MSCs and activated T cells, a predominant increase in the level of TNF-α (but not IFN-γ) was observed in the system. Therefore, the immunosuppression was predominantly iNOS-dependent. On the other hand, the use of the contactless model resulted in the initiation of the alternative program that required IFN-γ production and, therefore, used NO production for immunosuppression [69].

## ALTERNATIVE MECHANSIMS OF MSC-MEDIATED IMMUNOSUPPRESSION

The mechanisms responsible for the MSC-mediated neutralization of the activation of the cells of the immune system are not confined to only IDO and NO secretion. It has been shown that MSCs permanently express the inducible enzyme cyclooxygenase-2 (COX-2), which is responsible for the synthesis of prostaglandin E2 (PGE2) from arachidonic acid. PGE2 is a lipid that negatively affects T cell activation. Incubation of MSCs in the presence of blood lymphocytes results in a considerable increase in the PGE2 level in a culture [59, 75, 77, 78]. This may imply interaction between MSCs and T cells, leading to the enhanced synthesis of immunosuppressor molecules. Incubation of MSCs in the presence of IFN-γ and TNF-α causes a boost in the COX-2 expression level and PGE2 secretion, thus attesting to the fact that the production of this regulatory molecule can be controlled by the inflammation level [77]. The introduction of PGE2 inhibitors into a mixed culture consisting of T cells and MSCs resulted in a significant decrease in the immunosuppression level [77, 78].

It has been demonstrated that when incubated with lymphocytes or proinflammatory cytokines, MSCs secrete enhanced levels of IL-10 and TGF-β; anti-inflammatory cytokines that have a negative effect on the activation and division of T cells. The immunosuppressive effect that has been observed *in vitro* in the absence of antibodies can be partially eliminated by blocking antibodies against these cytokines [79]. It is believed that the secretion of IL-10 and TGF-β by activated MSCs accelerates the expansion of regulatory T cells, a minor population of CD4 lymphocytes, which are powerful negative immune response regulators, rather than just having a direct impact on T cells [80].

The nonclassical molecule of the histocompatibility complex class I antigen, G5 (HLA-G5), is another soluble factor that presumably participates in the MSC-mediated regulation of the immune response. Molecules of this type play a significant role in the establishment of immunological tolerance during pregnancy. The soluble HLA-G5 isoform is secreted by MSCs in the presence of contacts between MSCs and T cells in heterologous mixed cultures. HLA-G5 suppresses T cell proliferation and the cytotoxic properties of NK cells; simultaneously, it accelerates the division of regulatory T cells [65].

It has recently been established that MSCs express a set of Toll-like receptors (TLR), which are responsible for the recognition of the molecular patterns of various pathogens and innate immunity cell activation [81]. A MSC culture expresses a whole set of TLR (TLR1–TLR8) [82]. Stimulation of MSCs by incubating them with ligands of various TLR (such as LPS) results in the translocation of the NF-κB transcription factor to the nucleus and activation of the program, which simultaneously enhances the immunosuppressive properties of MSCs and increases IL-6 secretion in most cases [83, 84]. An increase in MSC activity upon TLR ligation can be easily accounted for by the fact that signal transduction pathways from the IFN-γ receptor and TLR intersect [83, 84]. Thus, the effect of TLR ligation may result (similarly to that for IFN-γ) in increased secretion of PGE2 and IDO [81].

The aforementioned mechanisms of MSC activation and immunosuppression are mediated by soluble factors. Meanwhile, mechanisms of MSC-mediated suppression of the immune response that depend on intercellular contacts have been described. One of the most well studied examples is the cell adhesion molecules ICAM-1 and VCAM-1 [85, 86], whose level on the surface of an MSC increases significantly in the presence of inflammation factors. These molecules are responsible for directed leukocyte migration and their penetration of the walls of blood vessels. It has been shown that an enhancement of the MSC-mediated expression of ICAM-1 and VCAM-1 is one of the possible immunosuppressive mechanisms, since the use of blocking antibodies against these molecules has reduced the level of MSC-mediated immunosuppression in a culture [85]. The results of experiments using cultures were supported by the data of *in*   *vivo* experiments, in which MSCs with the ICAM-1 and VCAM-1 genes knocked out were used for immunosuppression [85]. Unfortunately, unambiguous interpretation cannot be made of the results of these experiments, since the nonspecific contribution of the genetic defect to cell mobility cannot be distinguished from the direct contribution of ICAM-1 and VCAM-1 to the suppression of the T cell function.

## MSC-mediated immunosuppression *in vivo*

The ability of MSC to suppress the immune response in the context of the entire organism *in*   *vivo * was first detected during skin grafting experiments on monkeys. Transplanted MSCs decelerated the development of the immune response to the graft [68]. Moreover, it turned out that MSCs can be used in case of severe GVHD reaction (graft versus host disease). The transplantation of MSCs to mice, in which the lethal GVHD reaction after bone marrow transfer had been observed, enhanced their survival rate [87, 88]. At the time of writing, the mechanisms responsible for the improvement in clinical presentation have not been reliably determined; they have been only partially characterized in additional experiments using animals. Thus, it has been demonstrated that IFN-γ-deficient T cells are unsusceptible to MSC-mediated suppression in the GVHD model. In this system, the pre-activation of MSC by IFN-γ resulted in a fivefold increase in the immunosuppressive properties of MSCs as compared with those of the control cells [87–90].

It is tempting to use the immunosuppressive effect of MSCs upon human autoimmune diseases, such as diabetes mellitus, arthritis, multiple sclerosis, and systemic lupus erythematosus. In the experimental autoimmune encephalitis (EAE) model, an analogue of multiple sclerosis in mice, systemic transplantation of MSCs to the affected mice prevented the development of inflammatory infiltrates (T and B cells, macrophages) and that of the demyelination process in the CNS; moreover, it reduced the response of T cells to MOG peptides which originate from myelin [91]. The medium in which MSCs had been cultured suppressed the activation of CD4+ T cells under EAE conditions by reducing STAT-3 protein phosphorylation [92]. Infiltration of CD4+ T cells into the spinal cord of MSC-transplanted mice and the level of proinflammatory TNF-α and IL-17 cytokines were reduced [91]. In another study, MSC transplantation from Balb/c mice to B57BL/6 recipients with pronounced EAE symptoms caused alleviation of symptoms, such as reduction in the infiltration of immune cells in the CNS and a decrease in the blood level of IFN-γ and IL-17 cytokines [93].

In the collagen-induced arthritis mouse model, systemic transplantation of MSCs from human adipose tissue considerably reduced the probability of disease progression and its severity. The levels of inflammation and Th1-type immune response significantly decreased. The injection of MSCs resulted in the suppression of the expansion of the antigen-specific cells synthesizing IFN-γ and IL-17 [94]. Moreover, the enhanced secretion of anti-inflammatory IL-10 cytokine in the draining lymph nodes adjacent to the inflamed joints, and an increased number of CD4+CD25+Foxp3+ regulatory T cells were observed [94]. MSCs responded to collagen by suppressing the *in vitro * activation and division of T cells obtained from patients with rheumatoid arthritis and enhancing IL-10 secretion by T cells [95]. Furthermore, MSCs stimulated the formation of regulatory T cells capable of suppressing the response of T cells to collagen and reducing the level of the enzymes that destroy the intercellular matrix in synovial cells [95]. However, the results of an independent study using an induced arthritis model demonstrated that transfer of an specific subpopulation of MSCs expressing the Flk-1 marker, on the contrary, results in enhanced arthritic manifestations due to increased IL-6 secretion and Th17-type differentiation [96].

In the case of acute renal failure, the introduction of MSCs led to a recovery of renal function through a reduction in the level of proinflammatory cytokines (IL-1β, TNF-α, IFN-γ) [97]. The participation of MSCs in the regulation of the progression of fibrosis has been studied in a case of acute renal failure in rats. Along with the decrease in the IL-6 and TNF-α levels, the introduction of MSCs resulted in a reduction in fibrotic changes and recovery of the renal function. Moreover, an enhancement of the level of anti-inflammatory cytokines was observed [98]. In an experimental model of pulmonary fibrosis, the level of lung inflammation was reduced by the introduction of MSCs, presumably due to the secretion of a IL-1 receptor antagonist [99]. Upon autoimmune diabetes mellitus type 1, disease progression in prediabetic NOD mice was checked through the allogenic transfer of MCSs, which enhanced the type II immune response [72, 99, 100]. The prevention of β-cell destruction, followed by the progression of diabetes, was achieved through a single intravenous injection of MSCs; this can be accounted for by the induction of regulatory T cells [99, 100]. When introduced to rats with streptozotocin-induced β-cell damage, culture-expanded bone marrow MSCs migrated to the pancreatic gland, increased the level of insulin secretion, and facilitated the normalization of the level of blood glucose [101]. Furthermore, an increase in the PDX-1 and insulin levels in the Langerhans islets was observed, which assumes β-cell activation in mice receiving MSCs [101].

## CONCLUSIONS

In conclusion, it should be noted that reassuring data concerning the potential in using MSCs and drugs based on the factors secreted by them in the therapy of autoimmune diseases and regenerative medicine is already available. The data above provide convincing evidence that the immunosuppressive potential of MSCs can be enhanced by incubating the cells with inflammation factors and cytokines. Moreover, there is a possibility of obtaining genetically modified MSCs with improved immunosuppressive characteristics. However, it should be remembered that the infeasibility of strict control of the state of MSCs in a culture and the insufficiently proved genetic stability of these cells obstruct the implementation of MSC-based cell technologies. The accumulation of data on the ability of MSC to support and accelerate tumor growth by secreting factors that positively impact tissue regeneration is another major reason for concern [102]. 

## Abbreviations

MSCs: multipotent mesenchymal stromal cells
CD: cluster of differentiation
SDF-1: stem cell-derived factor-1
CXCR4 C-X-C chemokine receptor 4; VEGF: vascular endothelial growth factor
IGF-1: insulin-like growth factor-1
BDNF: brain-derived neurotrophic factor
TGF-β: tumour growth factor
BMP: bone morphogenetic protein
IL-10: interleukin-10
TNF-α: tumour necrosis factor
NK: natural killers
DC: dendritic cells
IFN-γ: interferon gamma
MHC: major histocompatibility complex
IDO: indoleamine-2,3-dioxygenase
PGE2: prostaglandin E2
ICAM: intercellular adhesion molecule
VCAM: vascular cell adhesion protein
IL-1β: interleukin-1 beta
GVHD: graft versus host disease
EAE: experimental autoimmune encephalomyelitis
TLR: Toll-like receptor
HLA-G5: non-classical molecule of histocompatibility complex class I antigen, G5 isoform
